# 

**Published:** 1896-11

**Authors:** 


					﻿Vol. XVI.
NOVEMBER, 1896.
NO. 11.
THE
pOMlEOPATHlC pHY^lClAfl,
A Monthly Journal of Homœopathic Materia Medica
and Clinical Medicine.
“ He it a freeman whom truth make* free, and all are tlavet betide."
"Seek the Truth: come whence it may, coet what it will."
BDITBD AND PUBLISH BD BT
WALTER M. JAMES, M. E>.
PHILADELPHIA :
TSTo. 1231 LOCUST STREET.
LONDON:
ALFRED HEATH & CO., Sole Agents fob Gbeat Britain,
114 Ebury Street, Eaton Square.
Yearly Subeeription, $2.SO, invariably 4m advance. Single number*, 23 tit.
Entered at the Philadelphia Poet-OBee at Second-Clan Mail Matter.
				

